# Use and Application of mHealth Technologies in Perioperative Surgical Care: Narrative Review

**DOI:** 10.2196/52206

**Published:** 2025-10-10

**Authors:** Maria Camila Sierra, Henry To, Wan Jun Song

**Affiliations:** 1 Department of Surgery Werribee Mercy Hospital Werribee, Melbourne Australia; 2 Department of Medicine, Dentistry and Health Science University of Melbourne Melbourne Australia; 3 Medical Director Hospital in the Home at Monash Health Monash University Melbourne Australia; 4 Senior Researcher Monash University Melbourne Australia

**Keywords:** surgical specialties, general surgery, postoperative care, perioperative care, ambulatory monitoring, telehealth, telemonitoring, remote consultation, mHealth

## Abstract

**Background:**

Surgical procedures and their potential complications place substantial strain on patients, clinicians, and health care systems. These strains are driven by the anticipated morbidity and mortality, so that there is resource-intensive postoperative inpatient management. Given the concentration of surgical services within hospital settings, current standard levels of care have limitations such as communication gaps, time lapses before evaluation, and investment of resources, which limit accessibility and generate disparities in delivery of care. However, recent advances in digital health, including telemedicine platforms, mobile health (mHealth), and wearable technologies, present an opportunity to decentralize and extend perioperative care into community settings. This review explored how established mHealth technologies are being integrated into the perioperative pathway and their impact on surgical care delivery and outcomes. It also highlights possible emerging models of remote physician and patient interaction where benefits seem to be outweighing the risks.

**Objective:**

The aim of this narrative review was to present collected evidence for the use of established mHealth technologies in the surgical pathway of patients and highlight their readiness and potential in models of standard care.

**Methods:**

A comprehensive literature search was conducted across MEDLINE (via PubMed), Web of Science, and Scopus databases between October 2022 and May 2024. Additional sources were identified through reference list screening of relevant systematic reviews. Data were extracted and analyzed based on surgical specialty, type of mHealth intervention, cost-effectiveness, and ethical considerations. Findings were summarized in tables to illustrate key trends and variations across studies. The extracted data were tabulated and described qualitatively to highlight similarities, differences, and possible emerging trends across the studies.

**Results:**

A total of 28 articles published between 2008 and 2022 were included for qualitative analysis, with most (n=21, 75%) originating from the United States, Germany, and the United Kingdom. The study designs were predominantly randomized controlled trials (n=9, 32%) and observational studies (n=8, 29%). Collectively, these studies involved 6344 patients undergoing mHealth-based perioperative interventions primarily in general surgery, orthopedics, and oncology. Interventions frequently used smartphones (n=10, 36%) and wearable devices, often in combination with other tracking and measuring systems. Applications included wound monitoring, postoperative follow-up, and patient education. Data collection was multimodal and typically conducted daily, yet only 36% (10/28) of the articles reported defined follow-up periods. Cost-effectiveness was rarely assessed, with only 4% (1/28) of the articles reporting per-patient savings. Overall, 64% (18/28) of the articles were rated as low quality due to methodological limitations.

**Conclusions:**

mHealth- and telehealth-based interventions show promise in enhancing aspects of perioperative care by enabling remote monitoring, patient engagement, and improved care continuity. Future research should focus on scalable implementation, true cost-effectiveness analysis, equitable access, and integration into clinical workflows to ensure broad applicability in current models of care.

## Introduction

### Background

Surgical procedures and associated complications impose a significant burden on patients, treating teams, and health care systems that are related to the associated morbidity and mortality of the illness and treatment [1,2]. Consequently, there is a need for surgical management and follow-up to be conducted as inpatient care, where there is a wide availability of resources to attend to the circumstances in the patient surgical pathway. As procedures and aftercare are delivered at focal points (hospitals) where patients and resources are located, the number of patients assessed and treated is limited, and available resources vary greatly among institutions, cities, and countries, which places an important limitation on the standard of care worldwide [1-3].

The rapid progression of digital technology and artificial intelligence and the creation of new wireless wearable devices in recent years has initiated a transformation across numerous industries, and health care is now experiencing firsthand a transformation of its own [4]. Advances in mobile health (mHealth) technologies have allowed components of the care for patients to be adequately conducted away from the traditional hospital settings [5]. Telemedicine, mHealth, and similar technologies are a rapidly growing area where existing mobile technology is applied to aspects of standard care so that the same service can be provided in the community in a safe and tailored environment [5,6]. Despite innovations in digital technology, the surgical domain has understandably approached the integration and implementation of new and potentially revolutionary technologies with caution given the immediate and direct implications for patients’ safety [7]. There is an increasing overwhelming need for community-based postsurgical care to be provided in settings where there is a higher level of patient comfort and convenience and be tailored to the individual’s needs.

Given that the current standard of care for most surgical patients following hospital discharge does inherently present limitations such as communication gaps, time lapses before evaluation, and investment of resources, strategies in the perioperative setting can be analyzed at many levels to improve their efficiency and effectiveness. In this sense, mHealth strategies applied to the surgical patient pathway display great diversity in goal setting and applications, with hypothesized consequential improvements in patients’ perioperative well-being and management of health changes [8-10]. In addition, the redistribution of the service burden can inherently impact the health economic budget distribution positively by redirecting inpatient hospital resources to those in most need [8,10].

There is already a great deal of technologies used in the community setting that can be accessed for health needs [4-6]. Data networks already exist to create, access, and securely transfer data remotely from individuals or care centers. The ubiquitous use of smartphones and wearable devices already offers the opportunity for medical data to be collected and stored in a more reliable, agile, and effective way [11]. These technological conditions paired with the good digital literacy in the community enable digital education, transition, and uptake rates to be more feasible. There is some available evidence that demonstrates that highly complex tasks within the hospital, such as performing a surgical procedure and monitoring patients with acute complex conditions, can now be performed remotely in a safe manner [12]. Mobile technologies can also provide the technological link to a remote group of medical experts, increasing the interconnectedness and globalization of health care as well as access to specialist care in remote areas [13].

### Objectives

Given the readiness of the existing technologies and their potential, it is worth evaluating their application within the perioperative field. In this narrative review, we aimed to answer the questions of how already established mobile technologies (mHealth) can assist in the perioperative care of surgical patients, what their potential is for change in management, and what the different implementation possibilities are within the physician-patient interaction model. We collated research in which innovations were applied as interventions and outcomes were qualitatively analyzed.

## Methods

### Search Strategy

A comprehensive literature search was conducted between October 2022 and May 2024 across 3 electronic databases: MEDLINE (via PubMed), Web of Science, and Scopus. The search strategy was developed collaboratively by the study investigators and an academic research librarian to ensure methodological rigor.

The search combined controlled vocabulary terms (eg, MeSH [Medical Subject Headings]) and free-text keywords related to surgical specialties, general surgery, postoperative and preoperative care, ambulatory monitoring, telehealth, telemonitoring, telemedicine, remote consultation, mHealth, and digital health interventions. Synonyms and related terms were used in combination with Boolean operators (AND and OR) to broaden retrieval sensitivity. The search was limited to English-language publications from January 1, 2000, onward. The detailed search strategy for MEDLINE is provided in Multimedia Appendix 1. The search terms and syntax were adapted to Scopus and Web of Science using database-specific indexing systems and search functions.

In addition to database searching, the reference lists of relevant systematic reviews were screened to identify additional studies meeting the inclusion criteria.

### Study Selection

This narrative review followed the PRISMA (Preferred Reporting Items for Systematic Reviews and Meta-Analyses) guidelines. After duplicate removal, titles and abstracts were independently screened by 2 reviewers (HT and MCS) to attain the final cohort of studies. Full-text articles were then retrieved and assessed against predefined inclusion and exclusion criteria (Textbox 1). In cases in which abstracts were unavailable or the available data were insufficient to determine relevance, the full article was reviewed and cross-checked against the research question and the predetermined eligibility criteria. Disagreements during the screening process were resolved through discussion between the 2 main researchers or consultation with a third reviewer (JS) when deemed relevant.

**Inclusion and exclusion criteria.**

**Inclusion criteria**

- English-language studies
- Human participant studies
- Original (new) data presentation and analysis

**Exclusion criteria**

- Studies that required the clinical team to be present in real time for the device to function
- Articles in languages that were not English
- Studies that involved patients with chronic illnesses
- Literature review articles
- Animal studies
- Studies on intraoperative interventions
- Study protocols
- Conference abstracts for which data could not be obtained
- Studies on telementoring
- Studies on remote monitoring by clinicians rather than remote monitoring performed by the patients

### Eligibility Criteria

Eligible articles were required to be original research papers published in English reporting studies involving human participants and presenting primary data analysis. Studies were excluded when the publication was in a language other than English, involved populations with chronic illnesses, or based their research on animal models. Other types of evidence such as literature reviews, study protocols, or conference abstracts lacking accessible data or studies that focused mainly on intraoperative interventions were also not deemed appropriate for data analysis. Articles that described telementoring or studies that involved remote monitoring by clinicians rather than patients were also excluded. This review was restricted to publications made from the 2000s onward to capture advancements in mHealth technologies (Textbox 1).

### Quality Appraisal

Although this is a narrative review, quality assessment was conducted to evaluate the strength and rigor of the evidence within the domains of mHealth and telemedicine. This step was deemed essential due to the emerging nature of these technologies and their growing relevance to contemporary models of care and intervention. Quality appraisal was undertaken independently by the researchers using criteria such as study design, risk of bias, potential confounding, allocation concealment, selective outcome reporting, loss to follow-up, early study termination, and imprecision of results. The appraisal was conducted under the framework and principles outlined in the article *Grading quality of evidence and strength of recommendations in clinical practice guidelines* [14]. Any discrepancies in quality rating were resolved through discussion. In accordance with the PRISMA guidelines and standards for reporting, transparency, and reproducibility, a summary of the quality assessment is provided in Multimedia Appendix 2 [10,15-41].

### Data Extraction

Data were extracted from full-text articles using a standardized extraction form developed specifically for this review to ensure consistency throughout the data collection process. Extracted data included publication details, surgical subspecialty, study design, sample size, technology used, metrics assessed, data collection frequency, and follow-up duration. Additional data points included patient satisfaction, safety outcomes, complication rates, cost analyses, and ethical considerations.

Data extraction was carried out independently by the 2 primary reviewers (HT and MCS) using Microsoft Excel. Any discrepancies were addressed through discussion. No automation tools were used for data extraction purposes.

### Synthesis of Results

A narrative synthesis was undertaken to summarize the findings of the included studies. Results were grouped thematically based on surgical specialty, type of mHealth or telemonitoring technology used, cost-effectiveness, and ethical considerations. The extracted data were tabulated and described qualitatively to highlight similarities, differences, and possible emerging trends across the studies.

**Figure 1:**
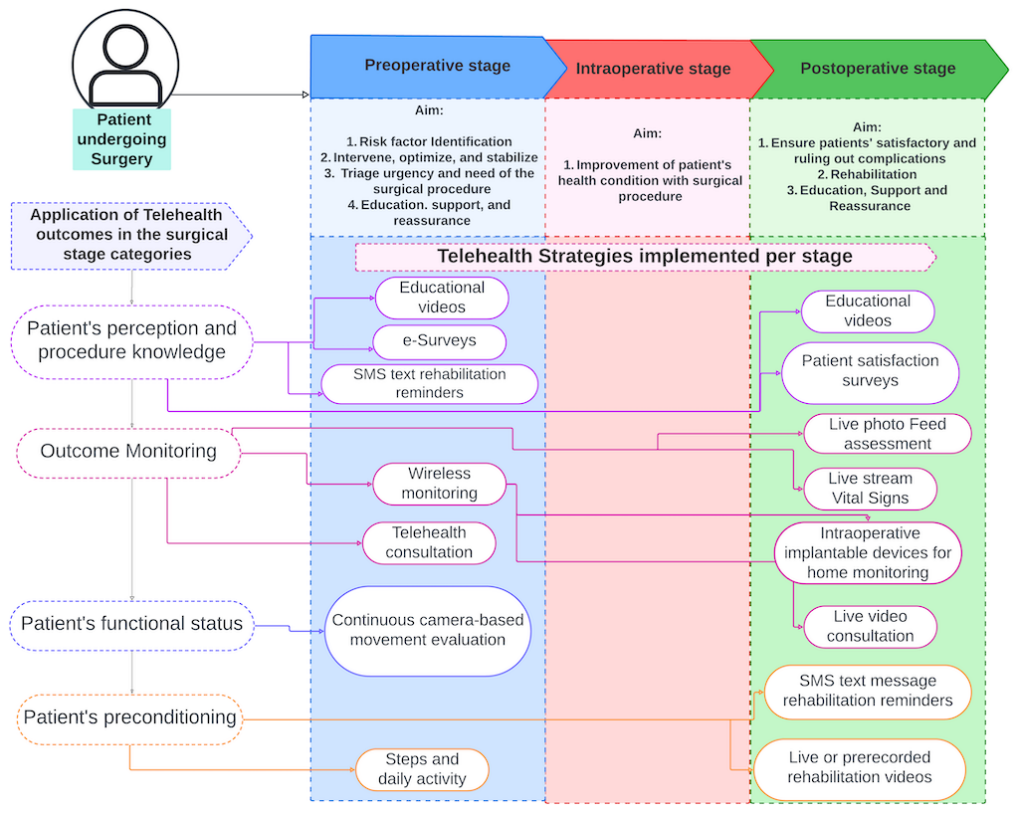
Patient surgical pathway (preoperative stage, intraoperative stage, and postoperative stage).

No meta-analysis was conducted due to the heterogeneity of study designs, outcome measures, and interventions. Where applicable, illustrative examples were provided to contextualize the findings and support interpretation (Figure 1).

## Results

### Study Selection

**Figure 2:**
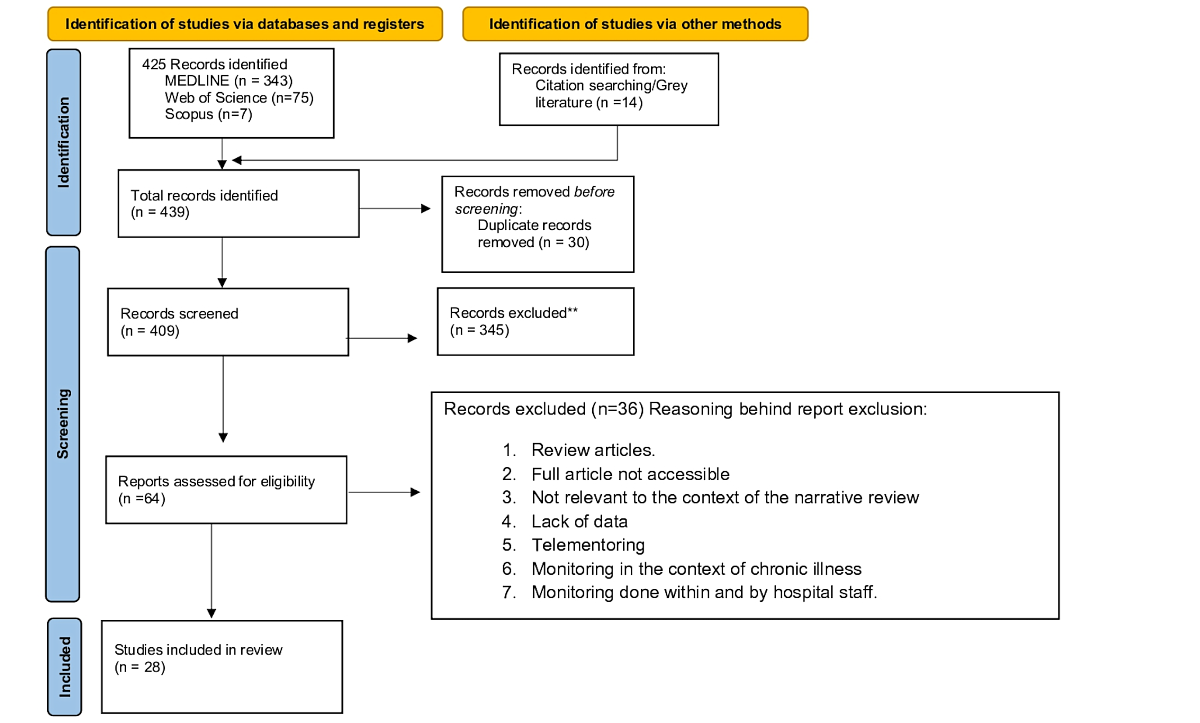
PRISMA flowchart describing the screening methodology for the articles selected for this review. **No automation tools were used; all references were screened by title and abstract by the two main researchers (MCS and HT). These articles were not deemed relevant based on the title and abstract. Image adapted from Page et al [42].

A total of 439 records were initially identified through database searching (n=425, 96.8%) and gray literature (n=14, 3.2%). After removal of 6.8% (30/439) of duplicates, 409 articles remained for title and abstract screening, from which 64 (15.6%) publications were deemed potentially relevant and underwent full-text review, as exemplified in Figure 2. Following eligibility assessment, 56% (36/64) of the articles were excluded due to lack of relevance to the narrative review, lack of accessible full text, insufficient data, focus on telementoring, or focus on monitoring in the context of chronic illnesses. A final selection of 44% (28/64) of the full-text articles was retained for data extraction [10,15-41]. A table with full disclosure of the selected references can be found in Multimedia Appendix 3 [10,15-41].

### Study Characteristics

The 28 included articles for data extraction were published between 2008 and 2022, and more than half (n=21, 75%) predominantly originated from the United States, Germany, and the United Kingdom. These countries had a publication upsurge from the year 2013 onward (Table 1).

Of the 28 included publications, 9 (32%) were randomized controlled trials, representing the highest level of evidence. Observational studies accounted for 29% (8/28) of the articles, and prospective and retrospective cohort studies each accounted for 4% (1/28) of the articles. Table 1 exhibits a trend toward higher levels of evidence in publications from 2018 onward when compared to previous years.

The included articles predominantly reported mHealth and telemonitoring strategies across 3 main specialties: general surgery, orthopedics, and oncology (Table 1). The most represented specialty was general surgery with 25% (7/28) of the studies, followed by orthopedics with 18% (5/28) of the studies and oncology with 11% (3/28) of the studies. Other specialties, including vascular surgery, obstetrics, procedural dermatology, and plastic surgery, each accounted for 7% (2/28) of the studies. Additional fields such as urology, transplant surgery, and nursing were each represented by 4% (1/28) of the studies.

**Table 1:** General characteristics of the included studies.

				Studies, n (%)
**Studies published in 2008-2012 (n=5)**				
	**Country of origin**			
		United States	3 (60)	
		Germany	1 (20)	
		Italy	1 (20)	
	**Surgical subspecialty**			
		Nursing	1 (20)	
		Plastic surgery	1 (20)	
		Microsurgery	1 (20)	
		Oncology	1 (20)	
		Vascular surgery	1 (20)	
	**Study design**			
		RCT^a^	2 (40)	
		Case-control	1 (20)	
		Observational study	1 (20)	
		Prospective cohort study	1 (20)	
	**Type of journal**			
		Clinical	5 (100)	
**Studies published in 2013-2017 (n=13)**				
	**Country of origin**			
		United States	9 (69)	
		Australia	1 (8)	
		Germany	1 (8)	
		United Kingdom	1 (8)	
		Spain	1 (8)	
	**Surgical subspecialty**			
		Orthopedics	4 (31)	
		General surgery	3 (23)	
		Dermatology	1 (8)	
		Oncology	1 (8)	
		Transplant	1 (8)	
		Neurosurgery	1 (8)	
		Vascular surgery	1 (8)	
		Vascular and general surgery	1 (8)	
	**Study design**			
		Observational study	6 (46)	
		Pilot study	2 (15)	
		Audit	1 (8)	
		Case series	1 (8)	
		Cross-sectional study	1 (8)	
		Retrospective cohort study	1 (8)	
		RCT	1 (8)	
	**Type of journal**			
		Clinical	13 (100)	
**Studies published in 2018-2022 (n=10)**				
	**Country of origin**			
		Germany	2 (20)	
		United Kingdom	2 (20)	
		United States	2 (20)	
		Canada	1 (10)	
		Iran	1 (10)	
		The Netherlands	1 (10)	
		The Philippines	1 (10)	
	**Surgical subspecialty**			
		General surgery	4 (40)	
		Obstetrics	2 (20)	
		Orthopedics	1 (10)	
		Plastic surgery	1 (10)	
		Urology	1 (10)	
		Dermatology	1 (10)	
	**Study design**			
		RCT	6 (60)	
		Case-control	1 (10)	
		Case series	1 (10)	
		Observational study	1 (10)	
		Systematic review	1 (10)	
	**Type of journal**			
		Clinical	7 (70)	
		Technology	2 (20)	
		Global health	1 (10)	
**Total articles included (n=28)**				
	**Country of origin**			
		United States	14 (50)	
		Germany	4 (14)	
		United Kingdom	3 (11)	
		Australia	1 (4)	
		Canada	1 (4)	
		Italy	1 (4)	
		Iran	1 (4)	
		The Netherlands	1 (4)	
		The Philippines	1 (4)	
		Spain	1 (4)	
	**Surgical subspecialty**			
		General surgery	7 (25)	
		Orthopedics	5 (18)	
		Oncology	3 (11)	
		Vascular surgery	2 (7)	
		Dermatology	2 (7)	
		Obstetrics	2 (7)	
		Plastic surgery	2 (7)	
		Microsurgery	1 (4)	
		Nursing	1 (4)	
		Transplant	1 (4)	
		Urology	1 (4)	
		Vascular and general surgery	1 (4)	
	**Study design**			
		RCT	9 (32)	
		Observational study	8 (29)	
		Case-control	2 (7)	
		Case series	2 (7)	
		Pilot study	2 (7)	
		Audit	1 (4)	
		Cross-sectional study	1 (4)	
		Prospective cohort study	1 (4)	
		Retrospective cohort study	1 (4)	
		Systematic review	1 (4)	
	**Type of journal**			
		Clinical	25 (89)	
		Technology	2 (7)	
		Global health	1 (4)	

^a^RCT: randomized controlled trial.

### Study Populations and Sample Sizes

Across the 28 studies, a total of 6344 patients participated in mHealth-related surgical research. Study cohorts varied widely in size, ranging from ≤50 patients to ≥1000 patients. As shown in Table 2, most studies had small sample sizes, with 46% (13/28) including ≤50 patients. In contrast, only 7% (2/28) of the studies recruited sample sizes as large as ≥1000 patients. Studies with intermediate sample sizes (51-500 patients) were the second most common type encountered in the collected evidence, representing 36% (10/28) of the selected studies.

Of the 28 articles deemed relevant, 14 (50%) reported the use of multidevice strategies for patient monitoring. These strategies combined smartphones, computers, tablets, smartwatches, and other unique wearable devices for outcome measurement (Table 2). Figure 1 provides a visual summary of the strategies assessed and implemented across the patient surgical pathway.

**Table 2:** Data features from the included studies (N=28).

Feature			Studies, n (%)
**Sample size**			
	≤50 patients	13 (46)	
	51-500 patients	10 (36)	
	501-1000 patients	3 (11)	
	≥1001 patients	2 (7)	
**Devices used for data collection**			
	Smartphone only	10 (36)	
	Tablet only	1 (4)	
	Computer only	0 (0)	
	Multidevice (combination of any of the aforementioned options or other external devices)	14 (50)	
	Not available	3 (11)	
**Parameters measured**			
	Vital signs	0 (0)	
	SSIs^a^	2 (7)	
	Patient satisfaction	3 (11)	
	Flap status and viability	2 (7)	
	Disability	0 (0)	
	Pain	0 (0)	
	Complications	0 (0)	
	Finances	1 (4)	
	Other (single option exclusive to a single study)	5 (18)	
	Multiple parameters	15 (54)	
**Metrics used**			
	Surveys or questionnaires	4 (14)	
	Photos	3 (11)	
	Scoring systems	2 (7)	
	Rating scales	0 (0)	
	Costs	1 (4)	
	Other (combination of any of the aforementioned options)	14 (50)	
	Not available	4 (14)	
**Follow-up period**			
	Before surgery	1 (4)	
	0-72 h after surgery	3 (11)	
	>72 h up to 1 mo after surgery	10 (36)	
	>1 mo-1 y after surgery	6 (21)	
	>1 y after surgery	0 (0)	
	Not available	8 (29)	
**Data collection frequency**			
	Daily or less	14 (50)	
	At least once a week or less	4 (14)	
	Consistently (fortnightly)	3 (11)	
	Not available	7 (25)	

^a^SSI: surgical site infection.

### Devices and Outcome Measurement

Several studies (5/28, 18%) focused on unique wearable devices for functional outcome measurement. Kayaalp et al [20], Chiang et al [26], and Calliess et al [32] validated new wearable-based gait kinematic analysis systems to assess functional recovery, implant longevity, and quality of life after total knee arthroplasty. Kayaalp et al [20] demonstrated that a new wearable system for knee function monitoring in healthy individuals showed comparable accuracy to that of motion capture systems, enabling postoperative mobility monitoring without restricting daily activities. Chiang et al [26] and Calliess et al [32] further supported the feasibility of wearable devices for continuous, objective assessment of functional outcomes.

The second most frequently reported mHealth strategy was the use of smartphones alone. Their use was reported in 36% (10/28) of the articles. Most smartphone-based interventions (10/28, 36%) revolved around web-based wound care and early detection of surgical site infections through photographic monitoring (3/28, 11%).

Henarejos et al [16] evaluated the effectiveness of an mHealth wound care education program in low- and middle-income countries using photographic monitoring to capture wound images during a 30-day follow-up. The images were shared with surgical teams via a digital exchange platform for timely intervention based on a color-coded classification system. The intervention resulted in a 19.1% reduction in surgical site infections despite study limitations associated with small sample size, loss to follow-up, and challenges related to health care infrastructure.

McLean et al [17] and Gunter et al [33] investigated smartphone-based models as assessment tools for early wound care, diagnosis, and intervention applied in the postoperative stage. McLean et al [17] focused on emergency abdominal surgery outcomes, including time of diagnosis, wound review attendance, and infection severity, whereas Gunter et al [33] focused on user satisfaction and validated a smartphone-based model as a wound monitoring system in vascular surgery. Both studies concluded that a smartphone-based model enabled postoperative follow-ups, early postoperative interventions, triage to appropriate care levels, and patient satisfaction.

Similarly, Hee Hwang and Mun [39] and Engel et al [15] explored smartphone-based models for remote free flap monitoring. Hee Hwang and Mun [39] demonstrated that smartphone-based flap assessment improved early diagnosis of flap compromise, increasing flap salvage rates from 96.2% to 100% and reducing reintervention times to 1.4 hours compared to a baseline of 4 hours. Engel et al [15] further validated the use of smartphone-based models as assessment systems and provided comparable accuracy rates, with shorter response times than those in in-person examinations.

Smartphones were also evaluated in the collection of data relevant to patient-centered outcomes, including rehabilitation, compliance, patient satisfaction, wound care reminders, and adherence to postsurgical rehabilitation programs. The studies by Hawkins et al [21], Segura-Sampedro et al [24], and Chee et al [28] appraised patient acceptance of smartphone-based models for follow-up. Segura-Sampedro et al [24] found that telemedicine-based follow-up services had a high sensitivity and specificity, supporting their feasibility and safety. Collectively, these studies reported reduced patient anxiety, improved postsurgical care understanding, and high acceptance of a self-monitoring program.

Although tablet-based interventions were less common, they were primarily used for patient satisfaction assessment and symptom tracking. Ertel et al [30] analyzed the feasibility of a video-based educational program and a telehealth monitoring model for post–liver transplantation care, including postoperative care management and readmission rates. The study concluded that the intervention was feasible, improved vital statistic maintenance, and enhanced patient awareness of preoperative and postoperative care management.

No articles identified in the literature search reported the use of artificial intelligence–based data capture, analysis, or monitoring.

### Data Collection and Metrics

As shown in Table 2, the studies used a wide and variable range of data collection methods, including surveys, questionnaires, photos, rating scales, and scoring systems. In total, 50% (14/28) of the studies used a combination of these metrics to assess patient-reported outcomes, satisfaction levels, attitudes toward mHealth interventions, home monitoring, and pain or disability.

For instance, Khoshrounejad et al [25] investigated the impact of an SMS text message–based rehabilitation program after corrective hand surgery using questionnaires and rating scales to measure variables such as pain, disability, physiotherapy adherence, and patient satisfaction. The intervention demonstrated increased motivation for self-directed rehabilitation. Nevertheless, reduced rehabilitation session attendance was noted.

The studies by Schramm et al [22], Sun et al [27], and Card et al [38] all used combined metrics for patient-centered outcomes using clinical measures. The study conducted by Sun et al [27] demonstrated that wireless monitoring programs involving real-time symptom tracking and mobility and quality of life assessment could be applied effectively among patients with cancer undergoing major abdominal surgery. Furthermore, wireless monitoring programs facilitated early intervention, enhancing perioperative care when applied across the patient surgical pathway (Figure 1) by detecting real-time changes in symptom severity, mobility, and quality of life.

Data collection frequency varied, with 50% (14/28) of the articles reporting near-daily data collection (Table 2), whereas 11% (3/28) of the articles reported medium-term data collection (more frequently). Measurement duration variability was dependent on the parameters measured; for example, symptom burden, disability, rehabilitation progress, and patient satisfaction were commonly assessed over longer follow-up durations.

Despite frequent data collection being reported in most articles, 29% (8/28) of the articles did not report a follow-up period, leading to the assumption that data collection ended at patient discharge. Among the 36% (10/28) of the articles that specified a follow-up time frame, monitoring ranged from 72 hours to 1 month after surgery.

### Economic Outcomes and Cost-Effectiveness

Only 7% (2/28) of the studies attempted to evaluate cost-related outcomes, with the study by Rosner et al [37] being the only one to systematically assess the financial impact of mHealth strategy application. The multicenter observational cohort study examined cost reductions in a 90-day period associated with an automated digital patient engagement platform in patients undergoing hip and knee arthroplasty. The intervention led to mean cost savings of US $656.52 per patient along with a relative reduction in 90-day readmission rates, hospitalization costs, and postoperative complications.

Barber et al [41] assessed telemetric intracranial pressure monitoring systems, which enabled home-based prolonged and safe intracranial pressure monitoring in neurosurgical patients. The system provided early reporting of combined outcomes across the preoperative and postoperative periods in the patient surgical pathway. Outcomes assessed included detection of clinical positive patient experiences, financial outcomes related to the outlay during pre- and postimplantation, and patient events. Clinical outcomes such as clinical events and associated complications were also deemed relevant for assessment. The system implementation yielded cost benefits by reducing unnecessary investigations.

### Quality of Evidence

The quality assessment of the selected studies is detailed in Multimedia Appendix 2. This was conducted by the main researchers individually, and 64% (18/28) of the selected studies were classified as low-quality evidence. Common methodological limitations included study design issues, lack of blinding, publication and selection bias, and small sample sizes, which limited the strength of associations. mHealth research is concentrated within certain surgical specialties and typically limited to a single stage of the patient’s pathway, mostly the postoperative period. As a result, its generalizability is limited, since findings cannot be easily applied across other specialties or the entire surgical pathway in different models of care.

## Discussion

### Principal Findings

#### Overview

Digital health care technologies, including mHealth strategies and telehealth, represent a rapidly evolving area where technology and health care intersect across the perioperative surgical care continuum. This narrative review highlights the significant opportunities that these technologies offer to enhance perioperative surgical care, particularly in the context of increasing health costs, limited inpatient resources, and the complexity of modern surgical procedures [43,44].

While telemedicine and remote consultation are already well integrated and show established benefits in clinical settings such as clinics, emergency departments, and rural trauma systems [15,45,46], this review demonstrated how successful digital health care technology implementation across the patient surgical pathway can be, impacting areas such as communication, patient convenience, and cost-effectiveness and care outcomes. The implementation of these technologies could streamline follow-up care and rehabilitation and significantly impact gaps in traditional surgical care, with the potential to substitute some components of inpatient care in a community setting.

#### Devices and Outcome Measurement

mHealth interventions often leverage common consumer technologies such as smartphones, tablets, smartwatches, and computers without requiring specialized or complex infrastructure. For instance, McGillicuddy et al [18] demonstrated how smartphones combined with wireless blood pressure monitors and medication trays supported postoperative care in patients undergoing transplants. Innovative approaches in the orthopedic field also showed promise, with 7% (2/28) of the studies developing and validating technologies for motion assessment in orthopedic care. These systems provided a cost-effective alternative to the gold-standard camera-based gait analysis systems using mobile technology, offering greater accessibility to patient condition assessment.

In the preoperative stage of the surgical patient journey, mHealth interventions improved new measuring strategies for patient engagement, assessment, and consistency and facilitated prehabilitation, as well as encouraging new modalities to provide informed consent and education. They also aided and positively influenced patients’ behavior, expectations, and compliance, aspects that highly influence the postoperative stage, potentially leading to future complications or truncated recovery if not handled adequately. Hawkins et al [21] found that tailored educational videos reduced procedural anxiety and improved wound care understanding. Similarly, Migden et al [47] reported that preconsent video education enhanced consultation efficiency and patient satisfaction. Furthermore, preoperative SMS text messages were shown to support medication adherence and treatment continuity [48,49]. The implementation of these strategies demonstrates how digital tools can positively influence patient outcomes and impact surgical ones.

Postoperative applications focused on remote monitoring, rehabilitation, and complication detection. Studies demonstrated benefits in patient-led rehabilitation, early complication detection through virtual wound care, functional recovery tracking via wearables, enhanced symptom reporting and triage, assessment of return to function and quality of life, and supporting patient education and self-management.

Considering the potential for communication gaps between patients and care teams during the first 2 to 3 weeks after patient discharge, interventions that facilitate detection of complications within the patients’ journey toward recovery, as well as early communication of rehabilitation modifications and recommencement of individual daily life activities, offer an opportunity for mHealth systems and groundbreaking technologies to be explored and applied. Several studies (18/28, 64%) highlighted the effectiveness of mHealth in this matter [50,51]. For instance, Khoshrounejad et al [25] found that SMS text message–based rehabilitation systems improved short-term hand function following tendon repair, whereas Chen et al [52] reported improved rehabilitation adherence and range of motion in patients with frozen shoulder who received motivational SMS text messages.

Wearable devices were particularly useful when assessing functional monitoring during early rehabilitation by offering synchronous and objective data collection. This approach addressed the often poor correlation between subjective self-report and actual physical activity, laying the groundwork for mHealth interventions to assist in more accurate, patient-centered recovery monitoring [29].

Wound care has emerged as a promising area for virtual monitoring, leveraging mobile device use to detect early complications and escalate standard care practices accordingly. Studies have shown that smartphone-based imaging models for wound assessment are equivalent to in-person examination even for complex wounds such as pressure ulcers [53]. Data highlight benefits such as travel time reduction, time-efficient triage, and greater health care provider productivity [53-55]. These findings support the plausible and potentially efficient integration of digital photography into standard wound care protocols [33].

#### Data Collection and Metrics

Multidevice systems improve measurement accuracy and response timeliness of patients to clinicians. McGillion et al [34] found that, although remote automated monitoring technology did not increase *days alive at home* after surgery, it enabled early identification and correction of medication errors, reduced pain levels, and lowered readmission and emergency visits within 30 days. These outcomes underline the acceptability of technological implementation in the context of virtual care by patients and physicians, as well as the potential cost-effectiveness of remote automated monitoring technology in postoperative care [34].

Finally, mHealth system implementation has also proven to positively impact quality of life and return to baseline function. Basch et al [35] demonstrated that web-based symptom reporting in oncology patients led to more timely interventions, improved symptom control, fewer emergency visits, and enhanced survival. This emphasizes how mHealth may augment clinician awareness and symptom management.

Despite the many promising findings, several challenges persist within this area of research. Many studies lacked standardized outcome measures, long-term follow-ups, or cost-effectiveness analyses. For example, Goode et al [36] reported no significant effects of mHealth interventions on postprostatectomy incontinence. This highlights the need for further and more robust research on the role of digital interventions in specific clinical contexts, particularly where in-person services are unavailable.

#### Economic Outcomes and Cost-Effectiveness

In terms of the cost-effectiveness of mHealth systems in surgery, it is clear that there is limited direct analysis available. Most of the existing data stem from inferred associations linking the benefits of mHealth systems to improved surgical outcomes rather than providing concrete cost-effectiveness evaluations. Notably, the study by Rosner et al [37] stands out as the only one in this review to directly explore and quantify the cost-effectiveness of mHealth application within the surgical context. Their findings revealed that patients using an automated digital patient engagement platform following hip and knee arthroplasty experienced a 54.4% reduction in potentially avoidable complications. This had direct financial implications, with the study reporting an average saving of US $656.52 per patient at 90 days after surgery [37].

#### Quality Assessment and Evidence Strength

mHealth systems and implementation of new technologies have several challenges to overcome as universal access and targeted assistance become a pressing priority within the field. This system implementation should be able to facilitate the creation of protocols that respond to the needs of diverse population groups, such as people with less digital literacy than the average population and people with special needs or difficulties when handling new devices due to their lack of familiarity with new technology. In this era of interconnectedness, stringent data security in mHealth apps and telemedicine is imperative, protecting patient security and private data. The implementation of these systems requires new strategies for patient confidentiality and data safeguarding to be optimized and improved. The creation of tools, wireless wearable devices, and other elements for mHealth and telemedicine application requires the active participation of health care professionals to direct objective assessment and fully fulfill the aims of the strategies, where protocol compliance should be top priority. Inherently, a formal cost-utility analysis is necessary as reimbursement for non–face-to-face care continues to be problematic under traditional fee-for-service payment models that may not yet include virtual assessment implementation [31].

While conducting this review, some gaps in the literature were identified. These included limited discussion on ethical and safety considerations regarding mHealth system implementation in the patient surgical pathway and few articles discussing how to upscale and roll out their interventions, as well as the implementation in different models of care. Furthermore, the data did not provide evidence suggesting the integration of mHealth systems into standard hospital-level care and operations, which, in conjunction with lack of devices, standardization, and follow-up timing standardization, jeopardizes the immediate implementation of these devices at a larger scale within the surgical field.

Regardless of the vast number of publications on the use of telemedicine, wireless wearable devices, and mobile apps in surgical specialties, the available data collected in this narrative review still need to be read and evaluated with caution as the collective impact continues to be varied and there seem to be substantial questions regarding the quality appraisal of the available evidence. When reading the available data, one must consider that studies are being conducted on a very small scale, compromising the possibility of establishing strong associations, which is a persistent limitation noted. Such limitations stress the need for multicenter trials and inclusive research that considers diverse health care contexts and infrastructure.

From the evidence gathered, none of the studies explored ethical considerations or system-level integration, making result generalizability an important aspect to consider in future research. Considering that the mHealth and telemedicine landscape is complex and varies from country to country, it is worth highlighting that most published articles and guidelines available are based on expert opinions, with evidence coming from high-income countries where strategies are applied to very specific scenarios and in highly controlled situations. Data then fail to provide insights on the challenges that may arise for low- and middle-income countries, where there is a substantial difference in circumstances, infrastructure, resource availability, and digital and educational literacy.

### Conclusions

Considering the context of a changing health care delivery system with a shift to community-based care, mHealth is aiding more creative patient monitoring methods in the outpatient setting. mHealth and telemedicine can be effectively implemented to actively engage patient populations, making individuals active participants in the process of monitoring their own conditions and actively involving them in the decision-making processes, which overall improves the experience, efficiency, and outcomes of care.

There is an increasing body of evidence pointing toward mHealth and telemedicine demonstrating significant promise across the surgical care field, but this evidence should be interpreted with caution. Many interventions have been evaluated in small, highly controlled studies, which limits generalizability. In addition, further research is needed to address implementation barriers; validate cost-effectiveness; and develop scalable, standardized frameworks applicable across health care systems in multiple settings and contexts. Future work should prioritize inclusive design, robust ethical safeguards, and equitable access, ensuring that digital solutions are effective, acceptable, and sustainable across diverse patient populations and care environments.

Despite the highlighted limitations, digital health technologies, including mHealth and telehealth interventions, show a considerable potential to improve perioperative surgical care. These tools’ strengths lie in enhancing patient education, enabling remote monitoring, supporting rehabilitation, and reducing unnecessary hospital visits, contributing to improved outcomes and system efficiency. Equally, there is evidence suggesting that mHealth technologies seem to have the potential to improve outcomes, reduce costs, and address gaps in perioperative care, particularly if future efforts prioritize equitable access, robust evaluation, and seamless integration into clinical workflows.

## Appendix

Multimedia Appendix 1 Full search strategy used for data queries in MEDLINE (via PubMed).

Multimedia Appendix 2 Quality appraisal table detailing the methodological evaluation of the included studies using the Grading of Recommendations Assessment, Development, and Evaluation framework.

Multimedia Appendix 3 Summary table of the selected studies, outlining authors, publication year, country where the study originated, journal, and specialty.

Multimedia Appendix 4 Evidence of ChatGPT (OpenAI) use during manuscript development, including prompts and artificial intelligence–generated content with author verification for language refinement purposes.

Multimedia Appendix 5 Evidence of ChatGPT (OpenAI) use during manuscript development, including prompts and artificial intelligence–generated content with author verification for formatting purposes for publication.

Multimedia Appendix 6 Supplementary summary tables created for the narrative review.

## Abbreviations

MeSH: Medical Subject Headings
mHealth: mobile health
PRISMA: Preferred Reporting Items for Systematic Reviews and Meta-Analyses

## Data Availability

Data sharing is not applicable to this article as no data sets were generated during this study. All data analyzed during this study is included in this published article and can be found in Multimedia Appendix 6 [10,15-41].
