# Supplementary material for: Use and Application of mHealth Technologies in Perioperative Surgical Care: Narrative Review
Source: JMIR Mhealth Uhealth. 2025 Oct 10;13:e52206. doi: 10.2196/52206 (PMC12552809; doi:10.2196/52206)
Supplement: Multimedia Appendix 1 [file mhealth_v13i1e52206_app1.docx]

# Multimedia Appendix 1. MEDLINE Full Search Strategy

| **Search Column #** | **Search Term** |
| --- | --- |
| **1** | exp General Surgery/ |
| **2** | exp specialties, surgical/ |
| **3** | exp post operative care/ |
| **4** | exp peri operative care/ |
| **5** | Limit 1 to English language |
| **6** | Limit 2 to English language |
| **7** | Limit 3 to English language |
| **8** | Limit 4 to English language |
| **9** | 5 OR 6 OR 7 OR 8 |
| **10** | Exp monitoring, ambulatory/ |
| **11** | Exp blood pressure monitoring, ambulatory/ |
| **12** | Exp monitoring, physiologic/ |
| **13** | Exp remote sensing technology/ |
| **14** | Exp wireless technology/ |
| **15** | Exp wearable electronic devices/ |
| **16** | Limit 10 to English language |
| **17** | Limit 11 to English language |
| **18** | Limit 1 2 to English language |
| **19** | Limit 13 to English language |
| **20** | Limit 14 to English language |
| **21** | Limit 15 to English language |
| **22** | 16 OR 17 OR 18 OR 19 OR 20 OR 21 |
| **23** | 9 AND 22 |
| **24** | Exp Telemedicine/ |
| **25** | Exp Remote consultation/ |
| **26** | Exp remote sensing technology/ |
| **27** | Exp Telemetry/ |
| **28** | m*health.mp. |
| **29** | Limit 24 to English language |
| **30** | Limit 25 to English language |
| **31** | Limit 26 to English language |
| **32** | Limit 27 to English language |
| **33** | Limit 28 to English language |
| **34** | 29 OR 30 OR 31 OR 32 OR 33 |
| **35** | 23 AND 34 |
