# Supplementary material for: Use and Application of mHealth Technologies in Perioperative Surgical Care: Narrative Review
Source: JMIR Mhealth Uhealth. 2025 Oct 10;13:e52206. doi: 10.2196/52206 (PMC12552809; doi:10.2196/52206)
Supplement: Multimedia Appendix 2 [file mhealth_v13i1e52206_app2.docx]

# Multimedia Appendix 2. Quality Appraisal Table.

| ***AUTHOR*** | ***SPECIALTY*** | ***QUALITY ASSESSMENT*** |
| --- | --- | --- |
| *Patricia S. Goode Et Al* | Urology | Moderate |
| *Farnaz Khoshrounejad Et Al* | Plastic Surgery | Low |
| *Valentin Henarejos Et Al* | General Surgery | Low |
| *Haveman Et Al* | General Surgery | High |
| *Kenneth A. Mclean Et* | General Surgery | High |
| *Michael McGillion Et Al* | General Surgery | Moderate |
| *Mahmut Enes Kayaalp Et Al* | Orthopaedics | Very Low |
| *Katharina Schramm Et Al* | Obstetrics | Low |
| *Alexandra E. Cairns Et Al* | Obstetrics | Low |
| *Spencer Hawkins Et Al* | Dermatology | Moderate |
| *Juan Jose Segura Sampedro Et Al* | General Surgery | Low |
| *Chih-Yen Chiang Et Al* | Orthopaedics | Low |
| *Virginia Sun Et Al* | Oncology | Low |
| *Benjamin Rosner Et Al* | Orthopaedics | Low |
| *Barber Et Al* | Neurosurgery | Low |
| *Shien-Ning Chee Et Al* | Dermatology | Low |
| *Ethan Basch Et Al* | Oncology | High |
| *Rebecca Gunter Et Al* | Vascular and General Surgery | Low |
| *Pa Toogood Et Al* | Orthopaedics | Low |
| *Audrey E. Ertel* | Transplant | Low |
| *Kristy Kummerow Broman Et Al* | General Surgery | Low |
| *Tilman Calliess Et Al* | Orthopaedics | Low |
| *John William McGillicuddy Et Al* | Vascular Surgery | Very Low |
| *Elizabeth Card Et Al* | Nursing | Very Low |
| *Jin Hee Hwang Et Al* | Plastic Surgery | Low |
| *Holger Engel Et Al* | Microsurgery | Moderate |
| *Charles S Cleeland Et Al* | Oncology | Low |
| *Domenico Palombo Et Al* | Vascular Surgery | Low |
