# Supplementary material for: Use and Application of mHealth Technologies in Perioperative Surgical Care: Narrative Review
Source: JMIR Mhealth Uhealth. 2025 Oct 10;13:e52206. doi: 10.2196/52206 (PMC12552809; doi:10.2196/52206)
Supplement: Multimedia Appendix 3 [file mhealth_v13i1e52206_app3.docx]

# Multimedia Appendix 3. Summary of Included References

*Table No. 1. Summary of Included Articles.*

| *Author* | *Year of Publication* | *Country* | *Journal* | *Specialty* |
| --- | --- | --- | --- | --- |
| *Patricia S. Goode Et Al* | 2022 | United States of America | Urology | Urology |
| *Farnaz Khoshrounejad Et Al* | 2022 | Iran | Hand Surgery Am | Plastic Surgery |
| *Valentin Henarejos Et Al* | 2022 | Philippines | Annals of Global Health | General Surgery |
| *Haveman*  *Et Al* | 2021 | Netherlands | Telemedicine and Telecare | General Surgery |
| *Kenneth A. Mclean Et Al* | 2021 | United Kingdom | Digital Medicine | General Surgery |
| *Michael Mcgillion Et Al* | 2021 | Canada | BMJ | General Surgery |
| *Mahmut Enes Kayaalp Et Al* | 2019 | Germany | MDPI | Orthopaedics |
| *Katharina Schramm Et Al* | 2018 | Germany | Archives of Gynaecology and Obstetrics | Obstetrics |
| *Alexandra E. Cairns Et Al* | 2018 | United Kingdom | American Heart Association | Obstetrics |
| *Spencer Hawkins Et Al* | 2018 | United States of America | American Society for Dermatologic Surgery | Dermatology |
| *Juan Jose Segura Sampedro Et Al* | 2018 | Spain | Annals of Medicine and Surgery | General Surgery |
| *Chih-Yen Chiang Et Al* | 2017 | United States of America | MDPI / Open Access/ Sensors | Orthopaedics |
| *Virginia Sun Et Al* | 2017 | United States of America | JAMA Surgery | Oncology |
| *Benjamin Rosner Et Al* | 2017 | United States of America | Journal of Arthroplasty | Orthopaedics |
| *Barber Et Al* | 2016 | United Kingdom | British Journal of Neurosurgery | Neurosurgery |
| *Shien-Ning Chee Et Al* | 2016 | Australia | Australasian Journal of Dermatology | Dermatology |
| *Ethan Basch Et Al* | 2016 | United States of America | Clinical Oncology | Oncology |
| *Rebecca Gunter Et Al* | 2016 | United States of America | JMIR mHealth and U health | Vascular and General Surgery |
| *Pa Toogood Et Al* | 2016 | United States of America | The Bone and Joint Journal | Orthopaedics |
| *Audrey E. Ertel Et Al* | 2016 | United States of America | Surgery | Transplant |
| *Kristy Kummerow Broman Et Al* | 2015 | United States of America | J Am Coll Surg | General Surgery |
| *Tilman Calliess Et Al* | 2014 | Germany | MDPI / Open Access/ Sensors | Orthopaedics |
| *John William Mcgillicuddy Et Al* | 2013 | United States of America | Medical Internet Research | Vascular Surgery |
| *Elizabeth Card Et Al* | 2012 | United States of America | Nursing and Management | Nursing |
| *Jin Hee Hwang Et Al* | 2012 | United States of America | Plastic and Reconstructive Surgery | Plastic Surgery |
| *Holger Engel Et Al* | 2011 | Germany | Wiley Periodicals | Microsurgery |
| *Charles S Cleeland Et Al* | 2011 | United States of America | Clinical Oncology | Oncology |
| *Domenico Palombo Et Al* | 2009 | Italy | Annals of Vascular Surgery | Vascular Surgery |
