# Supplementary material for: Use and Application of mHealth Technologies in Perioperative Surgical Care: Narrative Review
Source: JMIR Mhealth Uhealth. 2025 Oct 10;13:e52206. doi: 10.2196/52206 (PMC12552809; doi:10.2196/52206)
Supplement: Multimedia Appendix 4 [file mhealth_v13i1e52206_app4.docx]

# Multimedia Appendix 4. ChatGPT assistance evidence 1. Refining language and JMIR results formatting.

***Re write this in a more formal way for a narrative review "The 28 included articles for data extraction were published between 2008 and 2022, and more than half of the evidence (21 of 28) collected was predominantly originated from United States of America, Germany, and the United Kingdom. These countries had a publication upsurge from year 2013 and onwards (Table No. 2)" (CS 28/03/2025)***

###### **ChatGPT said:**

Certainly! Here's a more professionally worded version suitable for a narrative review:

"The 28 articles included for data extraction were published between 2008 and 2022. Notably, over half of the studies (21 out of 28) originated primarily from the United States, Germany, and the United Kingdom. These countries demonstrated a marked increase in publication activity beginning in 2013 (see Table 2)."

Let me know if you'd like it to sound more formal or adapted to a specific journal style.

##### You said:

Adapt to to JMIR journal

###### **ChatGPT said:**

Certainly! Here’s the revised version tailored to the tone and style typically used in JMIR (Journal of Medical Internet Research) publications, which emphasize clarity, precision, and a professional tone:

"Of the 28 articles included for data extraction, publication dates ranged from 2008 to 2022. The majority of studies (21/28, 75%) originated from the United States, Germany, and the United Kingdom. A notable increase in publication output from these countries was observed starting in 2013 (Table 2)."

Let me know if you're aligning with a specific JMIR sub-journal (e.g., JMIR mHealth and uHealth, JMIR Mental Health) or need help with other sections like Methods or Results formatting.

##### You said:

Yes JMIR mHealth and do need help with Result formatting

###### **ChatGPT said:**

Updated saved memory

Perfect! JMIR mHealth and uHealth typically follows a structured, concise, and data-driven style in the Results section. Here's a general format and a sample based on your earlier content to help guide you:

### **Results**

#### **Study Selection and Characteristics**

A total of 28 studies were included in the final analysis, with publication years ranging from 2008 to 2022. The majority of studies (21/28, 75%) originated from the United States, Germany, and the United Kingdom. A notable increase in publication frequency from these countries was observed beginning in 2013 (Table 2).

[Optional, if relevant: Studies were primarily observational/experimental/systematic reviews (add type), and focused on areas such as (list themes or foci).]

### JMIR Tips:

- Use precise numerical reporting (e.g., proportions, percentages).
- Present comparisons and trends clearly.
- Refer to tables/figures directly (e.g., “Table 2,” “Multimedia Appendix 1”).
- Avoid overly interpretive language—keep interpretations for the Discussion section.

If you’d like, I can help draft or refine the full Results section based on your data (just share your draft or notes). Want to do that?

##### You said:

All done, thank you

**ChatGPT said:**

You're very welcome! 😊 I'm glad everything's set. If you need help with anything else — submission steps, cover letter, or future projects — just give me a shout. Good luck with your manuscript! 🚀

4o
