# Supplementary material for: Use and Application of mHealth Technologies in Perioperative Surgical Care: Narrative Review
Source: JMIR Mhealth Uhealth. 2025 Oct 10;13:e52206. doi: 10.2196/52206 (PMC12552809; doi:10.2196/52206)
Supplement: Multimedia Appendix 5 [file mhealth_v13i1e52206_app5.docx]

# Multimedia Appendix 5. ChatGPT assistance evidence 2. Formatting for publication purposes.

***Please explain and provide an example of PRISMA checklist Methods section synthesis of results where it asks to specify the methods used to present and synthesise results in a narrative review (CS 20/04/2025)***

###### **ChatGPT said:**

Great question! The **PRISMA 2020 checklist**—under the **Methods** section—includes an item titled **"Synthesis of results"** (Item 13d), which asks authors to:

**"Describe the methods used to synthesize results. If meta-analysis was performed, describe the model(s), methods to identify the presence and extent of statistical heterogeneity, and software used."**

Since you're working on a **narrative review**, not a meta-analysis, you would not include statistical models, heterogeneity testing, or meta-analytic software. However, PRISMA still expects you to **describe how you organized, summarized, and interpreted** the results across the included studies.

### ✅ What should be included for a narrative review under “Synthesis of Results”?

You should describe:

1. **How results were grouped** (e.g., by surgical subspecialty, type of technology, outcome category).
2. **How data were presented** (e.g., narrative synthesis, tables, summary descriptions).
3. **Any frameworks or conceptual models** used to guide the synthesis.
4. **Whether comparisons were made** across studies and how.

***Can you please show an example on how Multimedia Appendixes need to be uploaded into the JMIR platform for publication (CS 22/04/2025)***

###### **ChatGPT said:**

Absolutely! Here’s a clear overview of **how to prepare and upload Multimedia Appendices** for JMIR mHealth and uHealth, based on JMIR Publications' official submission guidelines.

### ✅ **JMIR Multimedia Appendix Preparation & Upload Guide**

#### 📁 **1. What is a Multimedia Appendix?**

In JMIR journals, a Multimedia Appendix refers to any supplementary material that complements the main manuscript. This includes:

- Detailed search strategies
- Quality appraisal tables
- Extended data tables
- Survey instruments
- Algorithms or protocols
- Figures, multimedia (e.g., audio/video), or extended methods

### 📝 **2. Formatting Guidelines**

Each appendix should be:

- **Labelled** as:
  **Multimedia Appendix 1**, **Multimedia Appendix 2**, etc.
  (both in the manuscript text and in the file name)
- **Titled** with a **brief but descriptive title**
  Example: "Multimedia Appendix 1. Quality appraisal of included studies using GRADE criteria"
- **Formatted as**:
  - Word (.docx), Excel (.xlsx), or PDF (.pdf) — for tables and textual content
  - MP4, JPEG, PNG, or GIF — for multimedia content

🧠 Pro tip: Use consistent numbering and reference each appendix in the manuscript body using parentheses.
For example:
“...details of the quality appraisal process are provided in Multimedia Appendix 1.”

### ⬆️ **3. Uploading in the JMIR Submission System**

During the submission process in the JMIR Editorial Manager system:

1. On the **File Upload** step, choose **"Multimedia Appendix"** from the dropdown menu.
2. Upload each appendix **as a separate file**.
3. Provide a **brief title and description** in the upload form.
   - Example:
     - **File label**: Multimedia Appendix 1
     - **Description**: Quality appraisal of included studies using GRADE criteria
4. In your main manuscript file, ensure you’ve:
   - Referenced each appendix by number
   - Described the content briefly where it’s cited

### 📌 Example Reference in Manuscript

“The full quality assessment of included studies is presented in Multimedia Appendix 1.”

“Search strings for all databases are available in Multimedia Appendices 1–3.”

If you’d like, I can help **create a properly formatted Word or Excel appendix** file now when you provide more information, and label it as Multimedia Appendix 1. Would you like to proceed with that?

##### You said:

No, thank you so much for the assistance

###### **ChatGPT said:**

You're very welcome! 😊 I'm really glad I could help. If you need anything else—whether it's revisions, formatting, or submission support—I'm here for you. Best of luck with your JMIR mHealth and uHealth manuscript! You've clearly put in a lot of thoughtful work. 🌟

***Can you please explain this and provide a clear example that is tailored to JMIR standards "Data Availability - Please add a section on Data Availability. -- We encourage authors to ensure that their data sets are either deposited in publicly available repositories (where available and appropriate) or presented in the main manuscript or additional supporting files whenever possible. For example, these can be added as a URL to a repository and a URL to code used, attached as a Multimedia Appendix, or submitted/published separately in JMIR Data (https://data.jmir.org/). A Data Availability statement should still be provided even if access is restricted. In this case, a reason should be provided and possible steps for requesting access could be provided. For example statements which may be applicable to your data type(s) and study design, visit:*** [***https://support.jmir.org/hc/en-us/articles/360030832631-What-is-your-data-sharing-policy-***](https://support.jmir.org/hc/en-us/articles/360030832631-What-is-your-data-sharing-policy-)***" (CS 29/05/2025)***

###### **ChatGPT said:**

The **Data Availability** section in a JMIR article is a required component that informs readers **where and how the underlying data from the study can be accessed**. This is part of JMIR's open science policy aimed at increasing transparency, reproducibility, and trust in published research.

### Key Points JMIR Expects in This Section:

1. **State whether the data are publicly available**, restricted, or not available at all.
2. **Provide links or DOIs** to where the data/code is hosted (e.g., GitHub, Dryad, Figshare, institutional repositories).
3. **If access is restricted**, explain why (e.g., privacy concerns, proprietary data) and provide a contact or process for requesting access.
4. **Mention if data are available as Multimedia Appendices** with the manuscript.
5. **If no data were generated or analyzed**, you must still state that.

### 🔍 Example of a Data Availability Statement Tailored to JMIR Standards:

**Data Availability**
The deidentified data sets generated and analyzed during this study are available in the Open Science Framework repository at https://osf.io/xyz123. The R code used for statistical analysis is available at <https://github.com/username/projectname>. Additional supporting files are provided as Multimedia Appendix 1.

Due to ethical restrictions and the sensitive nature of participant information, raw identifiable data are not publicly available but may be shared upon reasonable request from qualified researchers. Requests should be directed to the corresponding author and will require institutional review board (IRB) approval.

##### You said:

##### That would be all. Thanks

###### **ChatGPT said:**

You're very welcome! Good luck with your submission to JMIR mHealth and uHealth. If you need help with revisions or anything else down the line, feel free to reach out.
